# Annual Dynamic Changes in Lignin Synthesis Metabolites in *Catalpa bungei* ‘Jinsi’

**DOI:** 10.3390/metabo15080493

**Published:** 2025-07-22

**Authors:** Chenxia Song, Yan Wang, Tao Sun, Yi Han, Yanjuan Mu, Xinyue Ji, Shuxin Zhang, Yanguo Sun, Fusheng Wu, Tao Liu, Ningning Li, Qingjun Han, Boqiang Tong, Xinghui Lu, Yizeng Lu

**Affiliations:** 1 College of Agriculture and Biology, Liaocheng University, Liaocheng 252000, China; 2 Institute of Germplasm Innovation, Shandong Provincial Center of Forest and Grass Germplasm Resources, Key Laboratory of National Forestry and Grassland Administration on Conservation and Utilization of Warm Temperate Zone Forest and Grass Germplasm Resources, Jinan 250102, China; 3 College of Forestry, Shandong Agricultural University, Taian 271018, China

**Keywords:** annual dynamic changes, *Catalpa bungei* ‘Jinsi’, lignin, metabolome

## Abstract

**Background**: *Catalpa bungei* ‘Jinsi’ has excellent wood properties and golden texture, which is widely used in producing furniture and crafts. The lignin content and structural composition often determine the use and value of wood. Hence, investigating the characteristics of the annual dynamics of lignin anabolic metabolites in *C. bungei* ‘Jinsi’ and analyzing their synthesis pathways are particularly important. **Methods**: We carried out targeted metabolomics analysis of lignin synthesis metabolites using ultra-performance liquid chromatography–tandem mass spectrometry (UPLC-MS/MS) on the xylem samples of *C. bungei* ‘Jinsi’ in February, April, July, October 2022, and January 2023. **Results**: A total of 10 lignin synthesis–related metabolites were detected: L-phenylalanine, cinnamic acid, *p*-coumaraldehyde, sinapic acid, *p*-coumaric acid, coniferaldehyde, ferulic acid, sinapaldehyde, caffeic acid, and sinapyl alcohol (annual total content from high to low). These metabolites were mainly annotated to the synthesis of secondary metabolites and phenylpropane biosynthesis. The annual total content of the 10 metabolites showed the tendency of “decreasing, then increasing, and then decreasing”. **Conclusions**: *C. bungei* ‘Jinsi’ is a typical G/S-lignin tree species, and the synthesis of G-lignin occurs earlier than that of S-lignin. The total metabolite content decreased rapidly, and the lignin anabolism process was active from April to July; the metabolites were accumulated, and the lignin anabolism process slowed down from July to October; the total metabolite content remained basically unchanged, and lignin synthesis slowed down or stagnated from October to January of the following year. This reveals the annual dynamic pattern of lignin biosynthesis, which contributes to improving the wood quality and yield of *C. bungei* ‘Jinsi’ and provides a theoretical basis for its targeted breeding.

## 1. Introduction

*Catalpa bungei* is a tall deciduous tree in the *Catalpa* genus belonging to the family Bignoniaceae, which has been known as the “King of Woods” since ancient times. *C. bungei* ‘Jinsi’, an important variant, is widely cultivated in Shandong, Anhui, Henan, and other provinces [1], and is named for the golden-yellow color of the heartwood and the golden texture of the longitudinal cuts that resemble gold threads [2]. *C. bungei* ‘Jinsi’ is a fast-growing, precious artificial timber species [1]. It has the excellent characteristics of a straight trunk, tough and fine material, beautiful texture, resistance to humidity and corrosion, and so forth [3]. It is widely used in furniture, architecture, musical instruments, precision instruments, and so forth, and has significant potential for high-end wood applications.

The composition and quality of wood often determine its use and value [4]. Wood is mainly composed of lignocellulose, in which lignin is a phenolic polymer. Lignin is synthesized through the phenylpropanoid pathway and is second only to cellulose in the plant as a source of biomass energy, accounting for l5–36% of the dry weight of wood. It can improve the mechanical strength of the plant, facilitate the transport of water, and alleviate the effects of adverse stresses and other biological functions [5]. Lignin can be classified into three types based on the composition of the three main alcohol monomers, *p*-coumaryl alcohol, coniferyl alcohol, and sinapyl alcohol: *p*-hydroxyphenyl lignin (H-lignin), guaiacyl lignin (G-lignin), and syringyl lignin (S-lignin) [6].

Yang and Li [7] performed a metabolomic analysis. They found that the transition from sapwood to heartwood of *C. bungei* ‘Jinsi’ mainly involved the accumulation of lignin constituents and biosynthesis of chromogenic substances, whereas the longitudinal direction of the wood did not change regularly with the height of the tree. Yi [8] identified naphthoquinone metabolites (α-lapachone and its derivatives) as the main substances contributing to the color of the heartwood of *C. bungei* ‘Jinsi’. They are synthesized in the sapwood, reaching the peak in the sapwood–heartwood transition zone; their synthesis stops in the heartwood, followed by their gradual degradation. Xu et al. [9] excavated the regulatory genes related to the color of *C. bungei* ‘Jinsi’. They hypothesized that the *CbuCYP71D15* gene was associated with the synthesis of 1,4-naphthoquinone compounds and acted as a hydroxylating agent. Also, the expression of the *CbuCYP71D15* gene was much higher in sapwood than in the leaves, branchlets, and roots. As shown in these studies, it is evident that much research has been dedicated to understanding the mechanisms of color formation in *C. bungei* ‘Jinsi’, but studies on the regularity of lignin biosynthesis remain limited. Research has demonstrated that wood (xylem) formation is regulated by both temporal and spatial factors, representing a complex and dynamic process. This process primarily involves cell division, cell expansion, cell wall thickening, and programmed cell death [10]. Developmental and environmental changes can significantly influence the lignin biosynthesis process, thereby impacting wood yield and characteristics. For example, in temperate regions, seasonal climate changes lead to distinct patterns in the activity of the cambium. In spring and early summer, cambial activity is relatively high, with rapid cell division and expansion forming earlywood, while in late summer and autumn, cambial cell division slows, resulting in the formation of latewood [11]. Thus, wood has been recognized as a biomarker of environmental changes. However, the seasonal rhythms of lignin biosynthesis in *C. bungei* ‘Jinsi’ remain unclear, and the annual variations in lignin metabolites are still undocumented. The gene regulatory network controlling lignin synthesis remains poorly understood, and therefore, further in-depth research on the lignin biosynthesis pathway in *C. bungei* ‘Jinsi’ is necessary.

In this study, lignin synthesis metabolites were measured in different months of the year using targeted metabolomics techniques, focusing on the differences and annual dynamics of lignin synthesis-related metabolites in different months. The aim was to investigate the lignin synthesis pathway of *C. bungei* ‘Jinsi’ and provide a reference for improving its timber properties and resource utilization.

## 2. Materials and Methods

### 2.1. Experimental Site and Sample Collection

The experimental material *C. bungei* ‘Jinsi’ (resource number: CB2017LYZ01) was grafted from Luoning County, Henan Province, in the spring of 2017 and planted in the spring of 2018 at the Zaoyuan Conservation Repository of the Warm Temperate Zone Rare and Endangered Tree Species National Forest Tree Germplasm Resource Repository, Shandong Province Forestry and Grassland Germplasm Resource Centre (36°45.3′ N, 117°27′ E), located in Zhangqiu district, Jinan City, Shandong Province, at an altitude of 60 m. Six asexual lines were preserved in the *C. bungei* germplasm resources nursery, all healthy and free of pests and diseases. The site is located in the temperate monsoon climate zone, with an average annual temperature of about 15.0 °C and an annual precipitation of about 660 mm.

**Figure 1:**
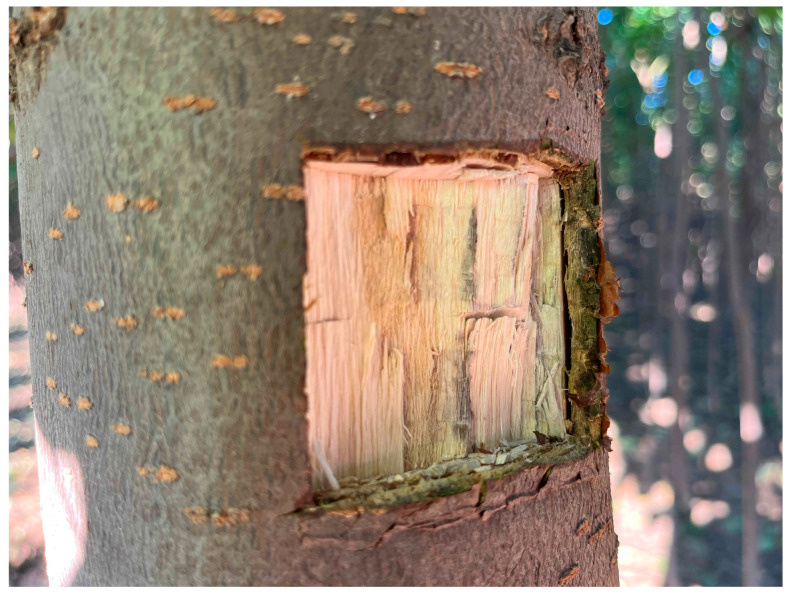
Sampling site of the xylem of *C. bungei* ‘Jinsi’.

The xylem samples were collected from the diameter at breast height of the *C. bungei* ‘Jinsi’ asexual line in February, April, July, October 2022, and January 2023. Each sample was taken from one asexual line, and four samples were collected as biological replicates each time. When taking samples, the bark was cut with a knife from 3 cm to 4 cm square, and then the exposed surface xylem was cut (Figure 1). The samples were wrapped with tinfoil, placed in liquid nitrogen for quick freezing, transported back to the laboratory, and placed in an ultra-low-temperature refrigerator at −80 °C. Each sample was grouped into one of five sample groups, as detailed in Table 1.

### 2.2. Methods

#### 2.2.1. Sample Extraction

After vacuum freeze-drying, the sample material was ground using a grinder (MM 400; Retsch GmbH, Haan, Germany) at 30 Hz for 1.5 min. Then, 100 mg of the powder was weighed and dissolved in 0.6 mL of 70% methanol aqueous solution. The dissolved samples were stored in a refrigerator at 4 °C overnight and vortexed six times during the process. They were then centrifuged at 10,000 rpm for 10 min. The supernatant was aspirated and then filtered with a micropore membrane (0.22-μm pore size) and stored in the injection bottle. The samples were then extracted using ultra-high-performance liquid chromatography (UHPLC, Shim-pack Ultra-Fast Liquid Chromatography, CBM-30A Data Acquisition and Control Module; Shimadzu, Kyoto, Japan) and tandem mass spectrometry (MS/MS, QTRAP 6500; Applied Biosystems, LLC, Foster City, CA, USA). The lignin-related standards (Sigma–Aldrich, Saint Louis, MO, USA) and acetonitrile, ethanol, and methanol (Merck, Darmstadt, HE, Germany) were chromatographically pure.

#### 2.2.2. UHPLC–MS/MS Conditions

The liquid chromatographic conditions were as follows: column: Agilent (Agilent, Santa Clara, CA, USA) SB-C18 1.8 µm, 2.1 mm × 100 mm; mobile phase: ultra-pure water (with 0.1% formic acid added) in phase A and acetonitrile in phase B; elution gradient: the proportion of phase B 5% at 0.00 min, increased linearly to 95% at 9.00 min, maintained at 95% for 1 min, decreased to 5% at 11.10 min, and equilibrated at 5% until 14 min; flow rate: 0.35 mL/min; column temperature: 40 °C; and injection volume: 4 μL.

The mass spectrometry conditions were as follows: electrospray ionization (ESI) at 550 °C; a mass spectrometry voltage of 5500 V; curtain gas (CUR) at 30 psi; collision-activated dissociation at a high, optimized declustering potential; and collision energy for scanning and detection.

#### 2.2.3. Data Processing

The primary and secondary mass spectrometry data were qualitatively analyzed based on the self-constructed database of Shanxi Bai’ai Gene Information Technology Co. Ltd., Shanxi, China, and the public databases of metabolite information, such as MassBank (http://www.massbank.jp/), KNApSAcK (http://kanaya.naist.jp/KNApSAcK/), HMDB (http://www.hmdb.ca/), MoTo DB (http://www.ab.wur.nl/moto/), and METLIN (http://metlin.scripps.edu/index.php), accessed on 1 May 2025. These databases were used to identify the metabolite structures. The quantitative analysis of metabolites was carried out using the multiple reaction monitoring (MRM) mode of triple quadrupole mass spectrometry. The peak area integration of the mass spectral peaks of all the substances was performed using MultiaQuant 3.0.3software after obtaining the metabolite spectral analysis data of different samples, and the mass spectral peaks of the same metabolite in different samples were corrected using integration correction [12]. The data related to mass spectra were processed using Analyst 1.6.3 software, and the data were organized using Excel 2021 software.

Subsequently, SPSS 26.0 software was used for the analysis of variance (ANOVA), along with principal component analysis (PCA), correlation analysis (CA), and hierarchical cluster analysis. Further, the fold change (FC) was calculated for each differential combination, and the P value was obtained using the Wilcoxon rank-sum test or *t* test, and FC ≥ 2 and FC ≤ 0.5 were used as the metabolite screening criteria to screen out the final differential metabolites. The corresponding differential metabolites were subjected to relevant pathway analyses using the Kyoto Encyclopedia of Genes and Genomes database.

## 3. Results and Analysis

### 3.1. Sample Quality Control Analysis

The quality control (QC) samples were prepared by mixing sample extracts. One QC sample was inserted for every 10 samples to detect and analyze mass spectrometry to monitor reproducibility in metabolite extraction and detection. The total ion current plots (Figure S1) of the QC samples showed high curve overlap, indicating consistent retention times and peak intensities. This demonstrated that the mass spectrometry results were stable and reliable, and the data from repeated analyses were consistent.

The qualitative and quantitative analyses of the metabolites in the samples were performed using mass spectrometry based on local metabolic databases. Each different-colored mass spectrometry peak in the MRM metabolite detection multi-peak plot represented a detected metabolite, and the peak area of each chromatographic peak represented the relative content of each corresponding substance.

### 3.2. PCA

**Figure 2:**
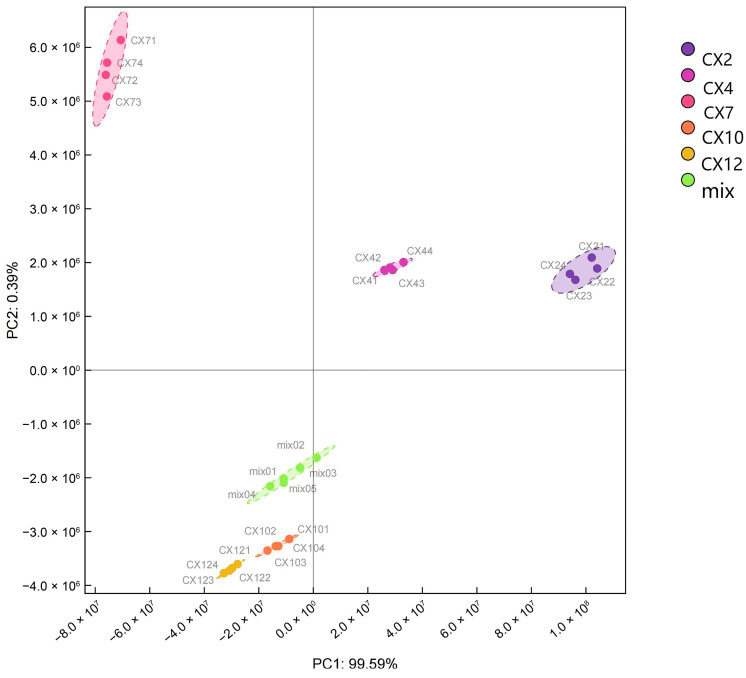
Principal component analysis of Metabolome samples.

The PCA model (Figure 2) was constructed for the 20 participating samples with principal component 1 (PC1) as the x-axis and principal component 2 (PC2) as the y-axis. The contribution rate of PC1 and PC2 was 99.59% and 0.39%, respectively. A significant separation trend was observed between different months. Also, the difference between different replicates of the same month was small and overlapping, indicating that the sample had good repeatability. Among these, PC1 separated February and April from July, October, and January 2023, and PC2 separated February, April, and July from October and January.

### 3.3. Qualitative and Quantitative Metabolite Analyses

#### 3.3.1. Analysis of Metabolite Composition in Different Months

The qualitative and quantitative analyses of lignin-related metabolites in each sample revealed the presence of 10 lignin-related metabolites (Table S1): L-phenylalanine, cinnamic acid, ferulic acid, sinapic acid, *p*-coumaric acid, caffeic acid, sinapaldehyde, *p*-coumaraldehyde, coniferaldehyde, and sinapyl alcohol. Among these, L-phenylalanine belonged to the class of amino acid and derivatives and accounted for the largest proportion of the total metabolites of the participating samples (about 91.63%), followed by phenylpropanoids, with a higher proportion of cinnamic acid (2.08%), *p*-coumaraldehyde (1.78%), sinapic acid (1.41%), *p*-coumaric acid (1.39%), and coniferaldehyde (1.03%). Ferulic acid and sinapaldehyde accounted for about 0.44% and 0.20%, respectively, whereas caffeic acid and sinapyl alcohol accounted for the least (only about 0.04% and 0.01%, respectively). Caffeic alcohol and caffeic aldehyde, related to C-lignin synthesis, were not detected.

**Figure 3:**
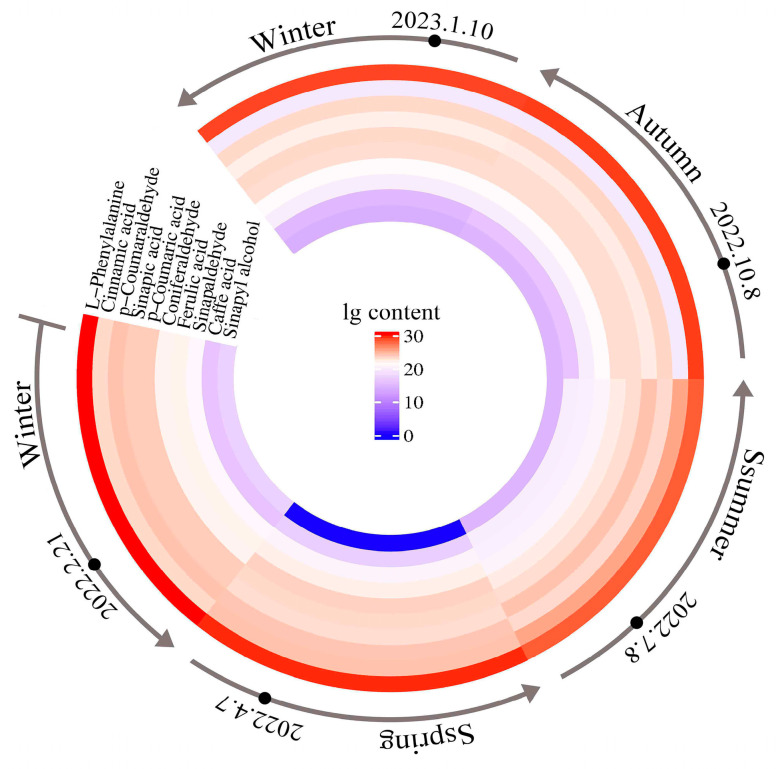
Dynamic changes in the annual contents of 10 metabolites.

The total content of the 10 metabolites in the samples showed a trend of “decreasing, then increasing, and then decreasing” between different months; the highest total content was found in February, and the lowest was found in July of that year (Figure 3). From February to July, the total content of the 10 metabolites decreased continuously, with a rapid decrease in the contents of precursor metabolites, such as phenylalanine, which drove lignin synthesis and metabolite conversion. From July to October, the total content of the 10 metabolites increased, and the intermediate metabolites of lignin synthesis gradually accumulated. Finally, from October to January of the following year, the total content of the 10 metabolites decreased slightly, indicating that lignin synthesis had slowed down or stagnated.

#### 3.3.2. Change Patterns of Metabolites in Different Months and ANOVA

**Figure 4:**
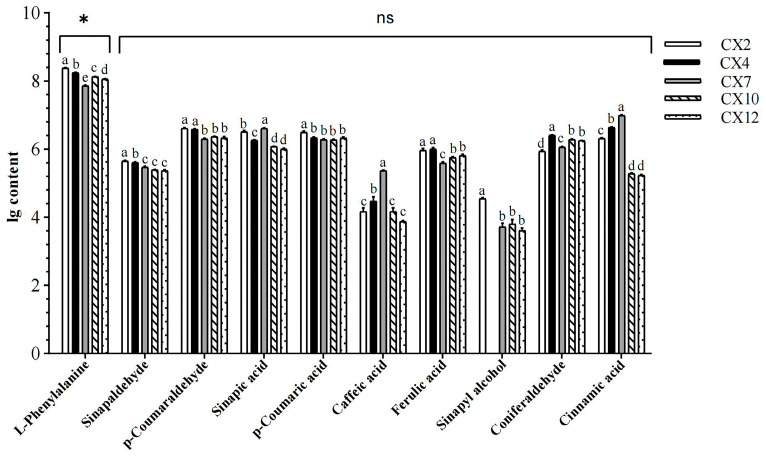
Changes in the contents of 10 metabolites in different months. Different lowercase letters indicate significant differences (*p* < 0.05); * indicates a significant difference at the 0.05 level, and ns indicates a nonsignificant difference at the 0.05 level.

ANOVA for lignin anabolites in different months revealed significant differences among the metabolites across different months (Figure 4, Table S2), with 10 metabolites showing varying synthesis and accumulation patterns over the course of the year. L-phenylalanine content always dominated. Among these, L-phenylalanine, *p*-coumaraldehyde, *p*-coumaric acid, and ferulic acid showed an overall trend of “decreasing, then increasing”, with the lowest content in July. Sinapic acid and sinapyl alcohol showed a trend of “decreasing, then increasing, and then decreasing”. Sinapic acid had the highest content in July, which was reduced by about 62.42% in January, and sinapyl alcohol showed a significant decrease in July compared with February. Coniferaldehyde showed a trend of “increasing, then decreasing, and then increasing”, with the content in April being about 2.3 times that in February. Caffeic acid and cinnamic acid showed a trend of “increasing, then decreasing”; in January, they reached 3.22% and 1.74%, respectively, of the content in July. Sinapaldehyde showed a decreasing trend. In addition, the maximum values of most metabolites were concentrated in February. Except for L-phenylalanine, the differences between the contents of other metabolites in October and January of the following year were not significant, indicating that the changes in the contents of the metabolites of lignin synthesis were small.

#### 3.3.3. Changes in Metabolite Accumulation Rates

**Figure 5:**
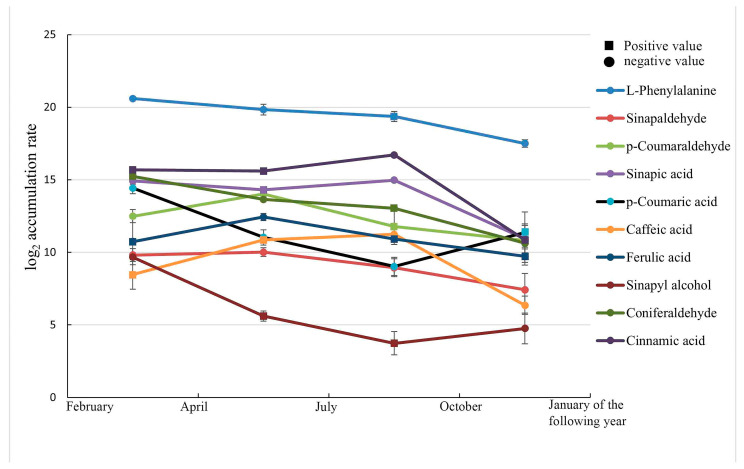
Changes in metabolite accumulation rate in different stages. The abscissa represents different time stages, and the ordinate is log_2_(accumulation rate). The accumulation rates in the figure indicate the speed of metabolite change (increase or decrease). The squares indicate a positive rate of metabolite accumulation, implying that the metabolite content increased during this period. The dots in the figure indicate the opposite.

The accumulation rates of each metabolite in different stages were calculated using the formula accumulation rate = | (W_n_ − W_n−1_)/time between two assays|, where W indicates the metabolite content (Figure 5). The results showed that the dynamic process of annual xylem metabolism of *C. bungei* ‘Jinsi’ was complex. Among the 10 metabolites, the accumulation rates of cinnamic acid, *p*-coumaraldehyde, ferulic acid, caffeic acid, and sinapaldehyde showed a trend of “increasing, then decreasing”, whereas the accumulation rates of L-phenylalanine and coniferaldehyde showed a “decreasing” trend. Also, the accumulation rates of *p*-coumaric acid and sinapyl alcohol showed a trend of “decreasing, then increasing”, and sinapic acid showed a trend of “decreasing, then increasing, and then decreasing”. The accumulation rate of each metabolite from October to January of the following year was low in 1 year, indicating that the lignin-related metabolism was relatively slow during this period. The metabolites changed relatively fast during the active growth period from April to October in summer and autumn, indicating active metabolism. In addition, L-phenylalanine and cinnamic acid had a significant accumulation rate, probably due to the involvement of various substances in the synthesis process, such as chlorogenic acid and flavonoid metabolites not specifically involved in lignin synthesis [13]. Therefore, the demand for their content was higher, and the change in accumulation rate was more complex. Sinapic acid, coniferaldehyde, *p*-coumaraldehyde, and ferulic acid also exhibited faster metabolic changes, indicating their importance as intermediate metabolites in lignin synthesis.

### 3.4. Correlation Analysis

**Figure 6:**
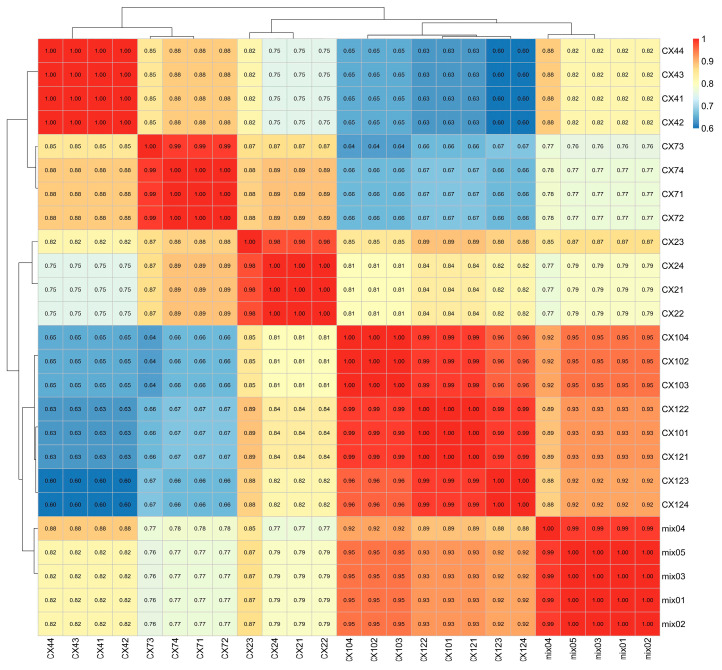
Correlation analysis of metabolic samples (*p* < 0.05).

The CA of the 20 participant samples (Figure 6) showed that the correlation coefficients were statistically significant (*p* < 0.05). Within the same group, the correlation coefficients were close to 1, indicating a strong correlation between the duplicate samples and a reliable selection of differential metabolites. In addition, CX10 and CX12 were highly correlated with each other, suggesting that the metabolites related to lignin synthesis were less changed, and the metabolic process was relatively slow from October to January of the following year. The correlation between February and April and between April and July was stronger, whereas the correlation between July and October and January of the following year was weaker. This indicated that July might represent an important turning point for lignin synthesis in *C. bungei* ‘Jinsi’, with significant changes likely occurring in the content and components of relevant metabolites during the period from July to October, a critical period for lignin synthesis.

### 3.5. Hierarchical Cluster Analysis

**Figure 7:**
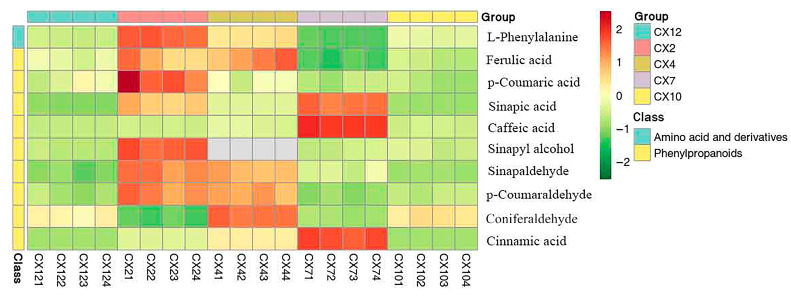
Cluster analysis of main related metabolites of lignin in different months.

The cluster analysis (Figure 7) showed that L-phenylalanine, *p*-coumaric acid, and sinapyl alcohol were more abundant in February, followed by sinapic acid, sinapaldehyde, ferulic acid, and *p*-coumaraldehyde. The contents of ferulic acid and coniferaldehyde increased, with coniferaldehyde showing a significant rise, whereas the contents of L-phenylalanine, sinapaldehyde, and *p*-coumaraldehyde decreased in April compared with February. The contents of sinapic acid, caffeic acid, and cinnamic acid increased significantly, whereas the contents of other metabolites decreased significantly in July. Most of the metabolites, especially caffeic acid, cinnamic acid, and sinapic acid, decreased significantly, whereas the content of coniferaldehyde increased slightly in October. The contents of metabolites basically did not change in January. These findings indicated that the metabolites related to lignin synthesis continuously underwent substance production and transformation from February to October. In contrast, the secondary metabolites of lignin synthesis were less active or involved from October to January of the following year.

### 3.6. Differential Metabolite Screening

#### 3.6.1. Statistical Analysis of the FC in Metabolites in Different Combinations

**Figure 8:**
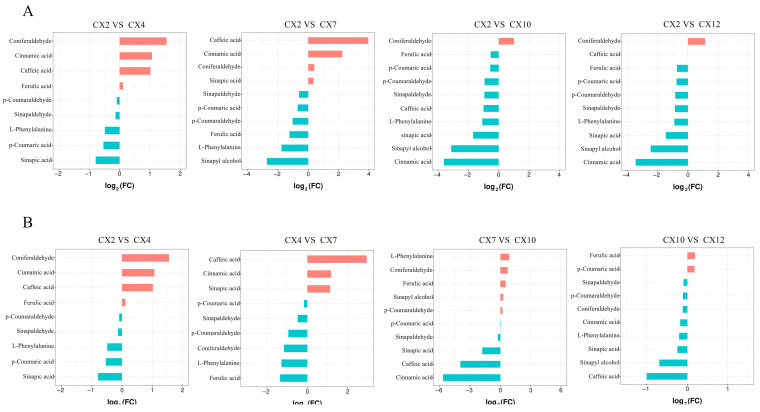
Statistics of FC in metabolites. (**A**) The difference in multiple statistics of metabolites in different combinations with February as the control; (**B**) the difference in multiple statistics of metabolites in different combinations with time sequence as control.

Taking February as the control, the FC of lignin synthesis metabolites in different combinations was compared and analyzed (Figure 8A). The upregulated and downregulated metabolites with the largest FC were coniferaldehyde and sinapic acid between February and April, caffeic acid and sinapyl alcohol between February and July, and coniferaldehyde and cinnamic acid between February and October and between February and January. According to the time sequence comparison (Figure 8B), the upregulated and downregulated metabolites with the largest FC were caffeic acid and ferulic acid between April and July, L-phenylalanine and cinnamic acid between July and October, and ferulic acid and caffeic acid between October and January.

Overall, the aforementioned analyses showed that the FC values in all nine metabolites except caffeic acid were small between October and January, and all of them showed an increase in coniferaldehyde content compared with that in February, suggesting that lignin synthesis in *C. bungei* ‘Jinsi’ was slower during this period. In addition, the use of February for control analysis (Figure 8A) more visually showed the differences between the contents of lignin synthesis-related metabolites and the initial metabolite contents in different months, as well as the changes in each metabolite in various stages. The comparative analysis in chronological order (Figure 8B) graphically shows the subtle dynamic changes in each metabolite between two adjacent time periods.

#### 3.6.2. Screening of Differential Metabolites in Different Months

The differential metabolites were screened using FC ≥ 2 and FC ≤ 0.5 as the criteria (Table 2). Differential metabolites were screened in all differential comparison combinations. The CX2 vs. CX7 and CX4 vs. CX7 combinations screened the most differential metabolites, both with six metabolites, with two upregulated and four downregulated in the former and three upregulated and three downregulated in the latter, indicating that the metabolites related to lignin synthesis underwent a large shift in lignin synthesis during this period. Further, only one differential metabolite was screened in the CX10 vs. CX12 combination, indicating that the metabolite changes were small and relatively stable during this period.

### 3.7. Analysis of Differential Metabolites

#### 3.7.1. Analysis of Differential Metabolites for Different Differential Combinations

As shown in Table 2, when February was used for the control analysis, the contents of coniferaldehyde, cinnamic acid, and caffeic acid were upregulated in April. The contents of caffeic acid and cinnamic acid were upregulated, whereas the contents of *p*-coumaraldehyde, ferulic acid, L-phenylalanine, and sinapyl alcohol were downregulated in July. The content of coniferaldehyde was upregulated, and the contents of sinapic acid, sinapyl alcohol, and cinnamic acid were downregulated in October. The content of coniferaldehyde was upregulated, and the contents of L-phenylalanine, sinapic acid, sinapyl alcohol, and cinnamic acid were downregulated in January. In contrast, when analyzed chronologically, the contents of caffeic acid, sinapic acid, and cinnamic acid were upregulated, whereas the contents of L-phenylalanine, coniferaldehyde, and ferulic acid were downregulated in July compared with April. Further, the contents of caffeic acid, cinnamic acid, and sinapic acid were downregulated in October compared with July, and the content of caffeic acid was downregulated in January compared with October.

The aforementioned analyses showed that the number of differential metabolites was higher from February to October and lower from October to January of the following year, suggesting that lignin anabolism continued from February to October and slowed or stagnated from October to January of the following year. The highest number of differential metabolites was found between April and July, and the number of differential metabolites decreased between February and April and between July and October. At the same time, the upstream metabolite L-phenylalanine was constantly consumed and converted into cinnamic acid from February to July, whereas the content of cinnamic acid was significantly reduced and lower than the content in February from July to October. It was speculated that July might be the key time point of lignin anabolism in *C. bungei* ‘Jinsi’, and the metabolism was more active before and after this period. However, the specific changes in the process need further investigation.

#### 3.7.2. Analysis of Common Differential Metabolites in Different Differential Combinations

**Figure 9:**
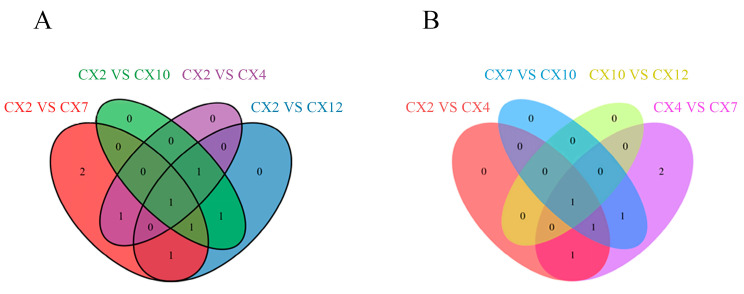
Venn diagram. (**A**) Venn diagram of differential metabolites in different combinations with February as control; (**B**) Venn diagram of differential metabolites of different combinations with time sequence as control.

The Venn diagram was drawn for different differential comparison combinations to further analyze the changes in differential metabolites and determine the common differential metabolites. Compared with February (Figure 9A), the Venn diagram was drawn for the four groups of CX2 vs. CX4, CX2 vs. CX7, CX2 vs. CX10, and CX2 vs. CX12, with only one common differential metabolite, cinnamic acid. According to the time sequence (Figure 9B), the Venn diagram was drawn for the four different combinations of CX2 vs. CX4, CX4 vs. CX7, CX7 vs. CX10, and CX10 vs. CX12, with only one common differential metabolite, caffeic acid. The comprehensive analysis showed that, except for the difference combination of CX10 VS CX12, the other nine different combinations had a common differential metabolite, cinnamic acid.

### 3.8. KEGG Enrichment Analysis

#### 3.8.1. KEGG Enrichment Analysis of Metabolites

Currently, 14 major metabolites are known to be involved in the lignin biosynthesis pathway [12]. Of these, 10 metabolites were detected in this study. KEGG functional annotation of these 10 metabolites (Table 3) revealed that cinnamic acid and caffeic alcohol were not annotated to the pathway, whereas the other 8 metabolites were annotated to 27 pathways. Among these pathways, those with the most annotated metabolites were the biosynthesis of secondary metabolites: ko01110 (8) and phenylpropanoid biosynthesis: ko00940 (8), followed by metabolic pathways—ko01100 (6) and the biosynthesis of phenylpropanoids—ko01061 (6). Additionally, caffeic acid and *p*-coumaric acid were annotated in the degradation of aromatic compounds—ko01220. L-phenylalanine was annotated in phenylalanine metabolism—ko00360, the biosynthesis of various plant secondary metabolites—ko00999, the biosynthesis of various other secondary metabolites—ko00997, and other pathways.

**Figure 10:**
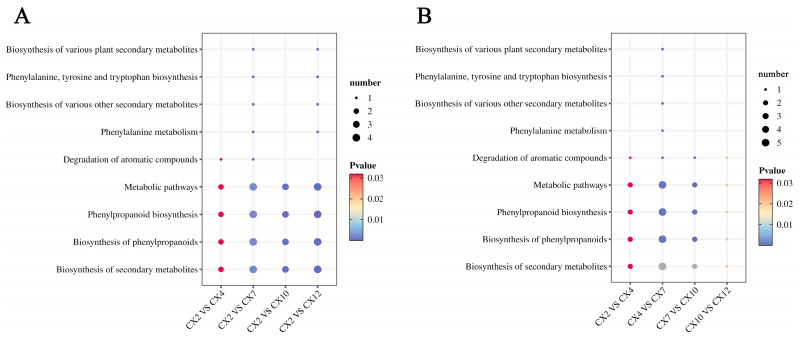
Comparison of KEGG enrichment analysis. (**A**) KEGG enrichment analysis of differential metabolites in different combinations with February as control; (**B**) KEGG enrichment analysis of differential metabolites in different combinations of time sequence control.

KEGG enrichment analysis of the differential metabolites of the different differential combinations revealed that most of the metabolites of the four differential combinations were annotated to the four pathways of the biosynthesis of secondary metabolite, phenylpropanoid biosynthesis, the biosynthesis of phenylpropane, and metabolic pathways, regardless of whether they were analyzed using February as the control (Figure 10A) or chronological control (Figure 10B). This further verified the participation of the detected metabolites in lignin biosynthesis, a primary metabolic process.

#### 3.8.2. Changes in Annual Dynamics of Lignin Anabolism

**Figure 11:**
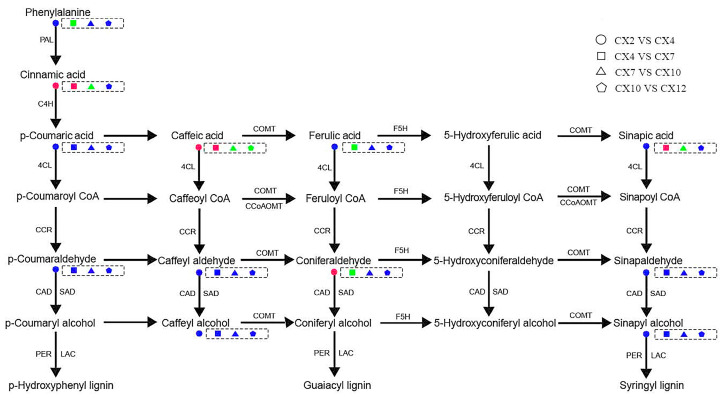
Annual dynamic changes in lignin synthesis pathway. Red represents upregulation of the metabolites, green represents downregulation of the metabolites, and blue represents insignificant differences.

The dynamics of metabolites in the lignin synthesis pathway of *C. bungei* ‘Jinsi’ were analyzed from February 2022 to January 2023 (Figure 11). The contents of cinnamic acid and caffeic acid were upregulated in April compared with February. The upstream part of the lignin synthesis pathway started to react in this period, whereas the content of the upstream metabolite of G-lignin monomer synthesis, coniferaldehyde, increased. The contents of cinnamic acid, caffeic acid, and sinapic acid were upregulated in July compared with April. This indicated that the synthesis pathway continued during this period and the initial metabolite, L-phenylalanine, was constantly consumed, downregulated, and converted into cinnamic and caffeic acid to provide sufficient precursor metabolites for the next monomer synthesis pathway. The content of the upstream metabolite, sinapic acid, used for the synthesis of S-lignin was upregulated, whereas ferulic acid and coniferaldehyde may be consumed, downregulated, and converted into coniferyl alcohol. Cinnamic acid and caffeic acid were consumed and downregulated in October compared with July, advancing monomer synthesis. Sinapic acid may be consumed, downregulated, and converted into sinapaldehyde. Lignin synthesis slowed down in October. Only caffeic acid was consumed and downregulated in January 2023 compared with October 2022, and the reaction was basically stagnant. These findings suggested that the synthesis of G-lignin may be earlier than that of S-lignin in *C. bungei* ‘Jinsi’.

## 4. Discussion

Lignification is a key evolutionary process in the transition of plants from lower to higher forms and from aquatic to terrestrial habitats, and wood is an important renewable resource and biomass energy source [14]. Therefore, lignin, as a major component of wood, has long attracted considerable attention. At present, the core pathway of lignin biosynthesis has been largely elucidated [15], encompassing monomer biosynthesis, transport, and polymerization, with all known lignin subunits derived from the phenylpropanoid metabolic pathway [16]. The metabolites detected in this study were similarly mainly annotated to the biosynthesis of secondary metabolites and the phenylpropanoid biosynthesis pathway (Figure 10), indicating that this is an essential metabolic route for lignin formation and illustrating the lignin deposition process. The lignin biosynthetic pathway has so far been shown to involve at least 13 genes encoding cytoplasmic enzymes, mediating 35 reactions and involving 25 metabolites [17]. This study identified 10 key metabolites involved in lignin biosynthesis in *C. bungei* ‘Jinsi’: L-phenylalanine, phenolic acids (cinnamic acid, ferulic acid, sinapic acid, *p*-coumaric acid, caffeic acid), phenylpropanoid aldehydes (sinapaldehyde, *p*-coumaraldehyde, coniferaldehyde), and phenolic alcohol (sinapyl alcohol). These metabolites exhibited significant variations in their concentrations throughout the annual dynamics of lignin biosynthesis. Among them, L-phenylalanine had the highest content, followed by such as cinnamic acid, *p*-coumaric acid, sinapic acid, and coniferaldehyde, while caffeic acid and sinapyl alcohol were the lowest. In addition, intermediates such as CoA esters and metabolites related to the branches producing catechyl lignin (C-lignin) and 5-hydroxyguaiacyl lignin (5H-lignin) were not detected (Figure 4). L-phenylalanine is the key initial substrate of the lignin biosynthetic pathway, converted to cinnamic acid by phenylalanine ammonia-lyase, with cinnamic acid further hydroxylated by cinnamate-4-hydroxylase to produce *p*-coumaric acid. These are critical precursor metabolites that determine the efficiency of downstream lignin biosynthesis, and they also participate in the biosynthesis of flavonoids and other secondary metabolites [12], showing a relatively high annual accumulation rate (Figure 5) and clear dynamic changes. Sinapic acid and coniferaldehyde are key rate-limiting metabolites for the biosynthesis of G-lignin and S-lignin, while sinapyl alcohol is the monomeric substrate used for S-lignin synthesis [16]. CoA esters, as intermediates in the lignin biosynthetic pathway, have short residence times and low stability, being rapidly and efficiently reduced to phenolic aldehydes by downstream enzymes, making them difficult to detect. Ultimately, lignin primarily polymerizes into H/G/S monomers, while C-lignin and 5H-lignin are considered non-canonical subunits that only naturally occur in certain species [18,19]. Overall, these 10 metabolites exhibited diverse annual dynamic patterns, with particularly large changes in accumulation rates from April to October, reflecting the complexity and diversity of the lignin biosynthetic process. Among them, cinnamic acid was the only common differential metabolite in nine pairwise comparisons (Figure 9), showing significant seasonal dynamics, suggesting that it may act as a key metabolite playing an important role throughout, while also potentially participating in the regulation of other physiological processes to enhance plant adaptability and respond in a timely manner to specific biological processes or environmental factors.

Wood formation is a seasonally driven, highly dynamic, and hierarchically regulated process [10], primarily influenced by environmental factors such as light [20], temperature [21], and water availability [22]. When these environmental conditions change—such as under high temperatures, drought, or intense light—it can significantly alter the lignin content, composition, or even trigger structural remodeling. Bang et al. [23] reported that drought stress in rice induces *OsNAC5*, which directly activates the *CCR10* gene, catalyzing the conversion of precursors into *p*-coumaraldehyde and coniferaldehyde, thereby promoting the deposition of H- and G-lignin. This process was also found to be accompanied by increased levels of abscisic acid [24] and jasmonic acid, along with a decrease in cytokinins [25]. Lima et al. [26] found that in *Coffea arabica*, high-temperature treatment led to increased contents of G- and S-lignin in leaves, whereas H-type lignin decreased. Falcioni et al. [27] showed that elevated gibberellin levels and enhanced light intensity significantly promoted xylem fiber wall thickening and lignin deposition in *Nicotiana tabacum* L. Consistent with these findings, the present study observed seasonal dynamic fluctuations in lignin biosynthetic metabolites. From February to July, increasing temperature and light intensity coincided with upregulated accumulation of precursor metabolites such as caffeic acid and cinnamic acid (Table 2), indicating an active lignin biosynthesis phase. From July to the following January, as temperature and light declined, the levels of caffeic acid and cinnamic acid decreased accordingly (Table 2), suggesting a gradual slowdown in lignin synthesis. Moreover, in the four pairwise differential comparisons relative to February (Figure 9A), July (summer) uniquely featured downregulated metabolites: *p*-coumaraldehyde and ferulic acid. This may reflect the stimulation of lignin biosynthesis by high temperature and drought in July, during which ferulic acid is rapidly consumed for G-lignin synthesis, enhancing mechanical stability and antioxidative capacity under stress [13].

Temperate tree species are capable of producing xylem cells with varying sizes, shapes, cell wall structures, and compositions across different seasons [28]. Lignin content and composition can vary significantly among different plant species, tissues, cell types, and even cell wall layers [15]. The annual metabolic process of lignin biosynthesis can generally be divided into five sequential developmental phases: reactivation, transition to active growth, active synthesis, transition to dormancy, and dormancy [29]. Accordingly, this study measured the contents of lignin biosynthetic metabolites in the sapwood of *C. bungei* ‘Jinsi’ across five months (February, April, July, October, and the following January), revealing a general trend of decrease–increase–decrease, with the lowest metabolite levels in July and the highest in February (Figure 3). Guo et al. [30] categorized the seasonal cycle of *Pinus tabuliformis* into three stages: active (June–July), transition (July–September), and dormant (September–March), and found the highest lignin content during the dormant phase, which aligns with our observations. In the current study, strong correlations were observed between October and January and between April and July (Figure 6 and Figure 7), reflecting distinct seasonal rhythmic patterns associated with winter and summer, respectively. During summer (the active phase), photosynthesis is strong, trees grow vigorously, and metabolic activity is high. However, high temperature and drought may limit enzyme activities in lignin biosynthetic pathways and constrain resource and energy supply. While stress may stimulate lignin biosynthesis to reduce water loss and resist adverse conditions, synthesis rates may be restricted, leading to increased metabolite consumption and decreased accumulation. In contrast, lignin biosynthesis slows down or stagnates in winter (dormant phase), but lignin content may still increase to reinforce mechanical strength and resist cold stress, though not throughout the entire winter period [31]. Furthermore, the elevated levels of certain metabolites in February suggest that although early spring remains part of the dormant phase, the reactivation of physiological processes likely begins in February [32], preparing for upcoming cambial activity. Similarly, temperate plants also exhibit differences between earlywood and latewood. Antonova et al. [33] found that in the lignification process of Scots pine, there was minimal variation in *p*-coumaric acid content, while *p*-coumaraldehyde gradually increased in earlywood and decreased in latewood. Ferulic acid significantly increased in latewood. Liszka et al. [34] observed that the lignin content in latewood of *P. sylvestris* was higher than that in earlywood, with a greater accumulation of coniferaldehyde in latewood. These findings are relatively consistent with the results of this study. During the formation of earlywood (April to July), the content of ferulic acid decreased, while the sinapic acid content increased. Regarding the formation of latewood (July to October), the ferulic acid content dramatically increased, while the sinapic acid content decreased. Additionally, there were minimal changes in *p*-coumaric acid content throughout the year. This could be attributed to the fact that during spring and early summer, temperatures rise, sunlight increases, and the water supply is ample. The cells are larger with thinner walls, leading to a higher content of sinapic acid and lower lignin deposition. In contrast, during late summer and autumn, temperatures drop, sunlight diminishes, and water supply becomes scarce. The precursor metabolite for G-lignin synthesis, coniferaldehyde, increases, the cells become smaller with thicker walls, and lignin deposition intensifies [35].

The types and contents of lignin vary among different plant species. Gymnosperms mainly contain G-lignin, dicotyledonous plants predominantly have G-S lignin, and monocotyledonous plants typically possess G-S-H lignin [36]. In this study, the synthetic precursors of H-lignin, namely, *p*-coumaric acid and *p*-coumaraldehyde, were detected, but coumaryl alcohol itself was not. Meanwhile, previous studies have found that the fiber cell walls of *C. bungei* contain almost no H-lignin [37], suggesting that *C. bungei* ‘Jinsi’ may lack H-lignin or only contain it in very small amounts. Among the 10 metabolites analyzed, L-phenylalanine, cinnamic acid, sinapic acid, *p*-coumaric acid, and coniferaldehyde showed relatively high contents, indicating that the lignin biosynthetic pathway in *C. bungei* ‘Jinsi’ is mainly directed toward the production of G- and S-lignin [38]. In addition, this study found that coniferaldehyde, a precursor of G-lignin, had a higher content and accumulation rate than sinapaldehyde, the precursor of S-lignin. This suggests that *C. bungei* ‘Jinsi’ is a typical G/S-lignin species, consistent with its material properties [7]. However, these characteristics may change over time or under different environmental conditions, and further research is needed to clarify this. In angiosperm cell walls, lignin monomers are generally deposited in the order of H-lignin, G-lignin, and S-lignin [14], but this process is influenced by external factors such as water availability and temperature [39]. Consistent with this, the present study observed that ferulic acid was significantly downregulated in July, likely reflecting its consumption for G-lignin synthesis, while sinapic acid was significantly downregulated in October, suggesting its consumption for S-lignin synthesis. Furthermore, as illustrated in the lignin biosynthesis pathway (Figure 11), the synthesis route for G-lignin is relatively short, whereas S-lignin requires additional hydroxylation and methylation steps. Therefore, G-lignin is preferentially deposited, promoting rapid cell wall construction, and it is inferred that in *C. bungei* ‘Jinsi’, the synthesis of G-lignin may occur earlier than that of S-lignin [40].

Lignin content and structure have a profound impact on wood quality and its end-use applications. When lignin content decreases below a certain threshold (around 40%), it can severely hinder the normal growth and development of transgenic plants [41]. Moreover, G-type monomers exhibit greater stability and structural integrity compared to S-type monomers, making their separation and degradation much more difficult [42]. *C. bungei* contains relatively abundant alcohol-benzene extractives, lignin, and hemicellulose, but has lower α-cellulose content and shorter fiber length, making it unsuitable for pulp and paper production, while being better suited for manufacturing high-end furniture and related applications [43]. Therefore, strategies to increase lignin content or reduce the S/G ratio in *C. bungei* ‘Jinsi’ have become important research directions. Currently, plants have developed a sophisticated regulatory network for lignin biosynthesis, in which environmental factors act in combination to induce a series of complex endogenous hormonal signaling pathways that activate transcriptional regulation (NAC and MYB transcription factors), enzyme activity modulation, and other processes to achieve lignin deposition and seasonal lignification remodeling [44]. While lignin metabolism exhibits considerable flexibility [15], the core biosynthetic pathway is highly conserved across most terrestrial plants, with all key genes for each step having been cloned [36]. PAL and C4H are rate-limiting enzymes at the initiation stage, controlling the flow of L-phenylalanine into the phenylpropanoid pathway. 4CL and C3H are located at branching points and influence the diversion of metabolites downstream. F5H, COMT, and CCoAOMT regulate hydroxylation and methylation processes, primarily determining the S/G ratio, while CCR and CAD are enzymes involved in specific reduction processes downstream, regulating both the total lignin content and structure [45]. It has been confirmed that the regulation of genes such as *CAld5H* and *COMT* can influence the S-lignin content, while genes like *CCoAOMT2* and *CCoAOMT3* can affect the G-lignin content. Additionally, genes such as *CCR2*, *PAL2*, *PAL4*, and *CAD1* can influence the overall lignin content [46]. Therefore, utilizing molecular biotechnology such as transgenics to modify the lignin content, composition, and intermediates or derivatives in the biosynthetic pathway of *C. bungei* ‘Jinsi’ can enable directed regulation and improve the quality and yield of its wood.

## 5. Conclusions

The 10 lignin synthesis precursor metabolites of *C. bungei* ‘Jinsi’ differed significantly among different months. From February 2022 to January 2023, the order of the total content from high to low was L-phenylalanine, cinnamic acid, *p*-coumaraldehyde, sinapic acid, *p*-coumaric acid, coniferaldehyde, ferulic acid, sinapaldehyde, caffeic acid, and sinapyl alcohol, and these metabolites were primarily annotated to the biosynthesis of secondary metabolites and the phenylpropanoid biosynthetic pathway. The total content of these 10 metabolites showed a trend of “first decreasing, then increasing, and then decreasing” in 1 year. It was also found that *C. bungei* ‘Jinsi’ was a typical G/S-lignin species, with G-lignin synthesis occurring earlier than that of S-lignin. Lignin synthesis and metabolism were more active from April to July, with metabolite accumulation from July to October, followed by a slowdown or stagnation from October to January 2023. This study reveals the dynamic variation characteristics of lignin biosynthetic metabolites in *C. bungei *‘Jinsi’ across different periods throughout the year and provides insights into its wood growth, development, and environmental adaptation features. These findings are of great significance for improving wood quality and yield and offer a theoretical basis for advancing molecular targeted breeding of *C. bungei* ‘Jinsi’ and promoting the efficient utilization of biomass resources.

## Figures and Tables

**Table 1:** Basic information of the xylem samples of *C. bungei* ‘Jinsi’.

Sample Location	Group	Sample Numbers	Acquisition Date
Xylem	CX2	CX21, CX22, CX23, and CX24	21 February 2022
Xylem	CX4	CX41, CX42, CX43, and CX44	7 April 2022
Xylem	CX7	CX71, CX72, CX73, and CX74	8 July 2022
Xylem	CX10	CX101, CX102, CX103, and CX104	8 October 2022
Xylem	CX12	CX121, CX122, CX123, and CX124	10 January 2023

**Table 2:** Screening statistics of differential metabolites in 10 differential comparison combinations.

Differential Comparison Combination	Differential Metabolites	Upregulated Expression of Differential Metabolites	Downregulated Expression of Differential Metabolites
CX2 vs. CX4	3	caffeic acid, coniferaldehyde, cinnamic acid	/
CX2 vs. CX7	6	caffeic acid, cinnamic acid	*p*-coumaraldehyde, L-phenylalanine, ferulic acid, sinapyl alcohol
CX2 vs. CX10	4	coniferaldehyde	cinnamic acid, sinapic acid, sinapyl alcohol
CX2 vs. CX12	5	coniferaldehyde	L-phenylalanine, cinnamic acid, sinapic acid, sinapyl alcohol
CX4 vs. CX7	6	sinapic acid, caffeic acid, cinnamic acid	L-phenylalanine, ferulic acid, coniferaldehyde
CX4 vs. CX10	2	/	caffeic acid, cinnamic acid
CX4 vs. CX12	2	/	caffeic acid, cinnamic acid
CX7 vs. CX10	3	/	sinapic acid, caffeic acid, cinnamic acid
CX7 vs. CX12	3	/	sinapic acid, caffeic acid, cinnamic acid
CX10 vs. CX12	1	/	caffeic acid

**Table 3:** KEGG annotation information of metabolites.

Metabolite	Classification	KEGG ID	Main KEGG Map
caffeic acid	Phenylpropanoids	C01197	ko01110, ko01061, ko00940, ko01220, and ko01100
coniferaldehyde	Phenylpropanoids	C02666	ko01110, ko01061, ko00940, and ko01100
cinnamic acid	Phenylpropanoids	C10438	–
L-phenylalanine	Amino acid and derivatives	C00079	ko01110, ko01061, ko00940, ko01100, ko00360, ko00997, ko00400, and ko00999
sinapic acid	Phenylpropanoids	C00482	ko01110, ko01061, ko00940, and ko01100
*p*-coumaric acid	Phenylpropanoids	C00811	ko01110, ko01061, ko00940, ko01220, and ko01100
ferulic acid	Phenylpropanoids	C01494	ko01110, ko01061, ko00940, and ko01100
sinapyl alcohol	Phenylpropanoids	C02325	ko01110, ko01061, ko00940, and ko01100
caffeic alcohol	Phenylpropanoids	C09066	–
caffeic aldehyde	Phenylpropanoids	C10945	ko00940 and ko01110
sinapaldehyde	Phenylpropanoids	–	–
*p*-coumaraldehyde	Phenylpropanoids	–	–

## Data Availability

All data are contained within the manuscript.

## Supplementary Materials

The following supporting information can be downloaded at: https://www.mdpi.com/article/10.3390/metabo15080493/s1, Figure S1: TIC overlap diagram of QC sample mass spectrometry detection; Table S1: Qualitative and quantitative lignin anabolite analysis table; Table S2: Quantitative analysis of variance for lignin anabolites.
