# Supplementary material for: Annual Dynamic Changes in Lignin Synthesis Metabolites in Catalpa bungei ‘Jinsi’
Source: Metabolites. 2025 Jul 22;15(8):493. doi: 10.3390/metabo15080493 (PMC12388274; doi:10.3390/metabo15080493)
Supplement: Supplementary file 1 [file metabolites-15-00493-s001.zip › metabolites-3677994-supplementary/Supplementary materials-Table S1.pdf]

Table S1. Qualitative and quantitative lignin anabolite analysis table.

Metabolite	Classification	CX21	CX22	CX23	CX24	CX41	CX42	CX43	CX44	CX71	CX72	CX73	CX74
L-Phenylalanine	Amino acid and derivatives	2.49E+08	2.51E+08	2.43E+08	2.41E+08	1.73E+08	1.75E+08	1.76E+08	1.80E+08	7.64E+07	7.09E+07	7.13E+07	7.14E+07
Sinapaldehyde	Phenylpropanoids	4.64E+05	4.64E+05	4.28E+05	4.45E+05	4.30E+05	4.09E+05	4.01E+05	4.02E+05	2.90E+05	3.01E+05	2.76E+05	3.16E+05
p-Coumaraldehyde	Phenylpropanoids	4.36E+06	4.21E+06	3.80E+06	4.03E+06	3.93E+06	3.81E+06	4.02E+06	3.63E+06	1.94E+06	2.12E+06	1.93E+06	2.04E+06
Sinapic acid	Phenylpropanoids	3.58E+06	3.18E+06	3.01E+06	3.24E+06	1.89E+06	1.89E+06	1.88E+06	1.87E+06	4.30E+06	3.95E+06	4.13E+06	4.16E+06
p-Coumaric acid	Phenylpropanoids	3.50E+06	3.05E+06	3.11E+06	2.90E+06	2.26E+06	1.97E+06	2.22E+06	2.23E+06	1.92E+06	1.83E+06	2.00E+06	2.00E+06
Caffeic acid	Phenylpropanoids	1.77E+04	1.20E+04	1.92E+04	1.18E+04	3.82E+04	3.87E+04	2.49E+04	2.14E+04	2.48E+05	2.32E+05	2.32E+05	2.33E+05
Ferulic acid	Phenylpropanoids	1.09E+06	9.62E+05	8.50E+05	8.43E+05	8.89E+05	9.87E+05	1.05E+06	1.12E+06	4.12E+05	3.64E+05	4.27E+05	3.88E+05
Sinapyl alcohol	Phenylpropanoids	3.82E+04	3.38E+04	3.53E+04	3.68E+04	N/A	N/A	N/A	N/A	4.48E+03	4.17E+03	6.18E+03	6.88E+03
Coniferaldehyde	Phenylpropanoids	9.23E+05	8.21E+05	9.58E+05	8.34E+05	2.67E+06	2.55E+06	2.57E+06	2.58E+06	1.20E+06	1.17E+06	1.13E+06	1.16E+06
Caffeic alcohol	Phenylpropanoids	N/A	N/A	N/A	N/A	N/A	N/A	N/A	N/A	N/A	N/A	N/A	N/A
Cinnamic acid	Phenylpropanoids	2.15E+06	2.04E+06	2.10E+06	2.16E+06	4.45E+06	4.46E+06	4.37E+06	4.47E+06	1.04E+07	9.94E+06	9.45E+06	1.01E+07
Caffeic aldehyde	Phenylpropanoids	N/A	N/A	N/A	N/A	N/A	N/A	N/A	N/A	N/A	N/A	N/A	N/A

Continued Table S1

CX101	CX102	CX103	CX104	CX121	CX122	CX123	CX124	Total CX2	Total CX4	Total CX7	TotalCX10	Total CX12	Total CX	比例%
1.38E+08	1.34E+08	1.30E+08	1.33E+08	1.19E+08	1.17E+08	1.14E+08	1.16E+08	9.84E+08	7.04E+08	2.90E+08	5.35E+08	2.51E+09	5.03E+09	91.3020%
2.50E+05	2.52E+05	2.52E+05	2.50E+05	2.39E+05	2.47E+05	2.19E+05	2.35E+05	1.80E+06	1.64E+06	1.18E+06	1.00E+06	5.63E+06	1.13E+07	0.2045%
2.32E+06	2.21E+06	2.40E+06	2.38E+06	2.41E+06	2.10E+06	1.96E+06	2.17E+06	1.64E+07	1.54E+07	8.03E+06	9.31E+06	4.91E+07	9.83E+07	1.7850%
1.24E+06	1.19E+06	1.16E+06	1.20E+06	1.10E+06	1.01E+06	9.67E+05	9.81E+05	1.30E+07	7.53E+06	1.65E+07	4.79E+06	4.19E+07	8.37E+07	1.5212%
2.01E+06	1.91E+06	1.83E+06	1.81E+06	1.95E+06	2.06E+06	2.35E+06	2.19E+06	1.26E+07	8.68E+06	7.75E+06	7.56E+06	3.66E+07	7.31E+07	1.3279%
1.49E+04	1.97E+04	1.53E+04	1.11E+04	7.60E+03	8.20E+03	7.62E+03	7.04E+03	6.07E+04	1.23E+05	9.45E+05	6.10E+04	1.19E+06	2.38E+06	0.0432%
6.11E+05	5.81E+05	5.49E+05	5.51E+05	6.89E+05	6.53E+05	5.93E+05	6.75E+05	3.75E+06	4.05E+06	1.59E+06	2.29E+06	1.17E+07	2.33E+07	0.4241%
8.45E+03	8.13E+03	4.52E+03	5.46E+03	5.38E+03	3.68E+03	3.69E+03	3.73E+03	1.44E+05	0.00E+00	2.17E+04	2.66E+04	1.92E+05	3.85E+05	0.0070%
1.86E+06	2.01E+06	1.96E+06	1.90E+06	1.81E+06	1.77E+06	1.71E+06	1.85E+06	3.54E+06	1.04E+07	4.66E+06	7.73E+06	2.63E+07	5.26E+07	0.9554%
N/A	N/A	N/A	N/A	N/A	N/A	N/A	N/A	N/A	N/A	N/A	N/A	N/A	N/A	N/A
2.02E+05	1.96E+05	1.92E+05	1.93E+05	1.74E+05	1.85E+05	1.66E+05	1.68E+05	8.45E+06	1.78E+07	3.99E+07	7.83E+05	6.69E+07	1.34E+08	2.4296%
N/A	N/A	N/A	N/A	N/A	N/A	N/A	N/A	N/A	N/A	N/A	N/A	N/A	N/A	N/A
