# Supplementary material for: Annual Dynamic Changes in Lignin Synthesis Metabolites in Catalpa bungei ‘Jinsi’
Source: Metabolites. 2025 Jul 22;15(8):493. doi: 10.3390/metabo15080493 (PMC12388274; doi:10.3390/metabo15080493)
Supplement: Supplementary file 1 [file metabolites-15-00493-s001.zip › metabolites-3677994-supplementary/Supplementary materials-Table S2.pdf]

Table S2. Qualit Analysis of variance for lignin anabolites.

Metabolite	Classification	CX2	CX4	CX7	CX10	CX12	mix
L-Phenylalanine	Amino acid and derivatives	246000000±4760952.29^{a}	176000000±2943920.29^{b}	72500000±2608958.92^{e}	133750000±3304037.93^{c}	116500000±2081666.00^{d}	138600000±6542170.89^{c}
Sinapaldehyde	Phenylpropanoids	450250±17327.72^{a}	410500±13478.38^{ab}	295750±16938.61b^{c}	251000±1154.70^{c}	235000±11775.68^{c}	410000±126739.89^{ab}
p-Coumaraldehyde	Phenylpropanoids	4100000±241246.76^{a}	3847500±168597.15^{a}	2007500±89953.69^{b}	2327500±85391.26^{b}	2160000±188148.88^{b}	2320000±196596.03^{b}
Sinapic acid	Phenylpropanoids	3252500±239078.09^{b}	1882500±9674.27^{c}	4135000±143874.95^{a}	1197500±33040.37^{e}	1014500±59746.69^{e}	1554000±174441.97^{d}
p-Coumaric acid	Phenylpropanoids	3140000±255734.24^{a}	2170000±134412.30^{b}	1937500±80983.54^{b}	1890000±90921.21^{b}	2137500±172312.70^{b}	2002000±200673.86^{b}
Caffeic acid	Phenylpropanoids	15175±3831.78^{d}	30800±8950.61^{c}	236250±7847.50^{a}	15250±3519.00^{d}	7615±473.67^{d}	72780±2737.15^{b}
Ferulic acid	Phenylpropanoids	936250±116098.74^{a}	1011500±98083.30^{a}	397750±27645.07^{c}	573000±29257.48^{b}	652500±42343.83^{b}	563000±44238.00^{b}
Sinapyl alcohol	Phenylpropanoids	36025±1898.03^{a}	NA	5427.5±1310.86^{c}	6640±1947.90^{c}	4120±840.28^{c}	19460±2553.04^{b}
Coniferaldehyde	Phenylpropanoids	884000±66997.51^{d}	2592500±53150.80^{a}	1165000±28867.51^{c}	1932500±66017.74^{b}	1785000±58721.60^{b}	2768000±182400.66^{a}
Caffeic alcohol	Phenylpropanoids	NA	NA	NA	NA	NA	NA
Cinnamic acid	Phenylpropanoids	2112500±55000.00^{c}	4437500±45734.74^{b}	9972500±397104.10^{a}	195750±4500.00^{e}	173250±8539.13^{e}	1446000±50299.11^{d}
Caffeic aldehyde	Phenylpropanoids	NA	NA	NA	NA	NA	NA

The data represent the mean±standard deviation of four replicates. Different lowercase letters indicate significant differences ($P < 0.05$).
