# Supplementary figures and images for: Annual Dynamic Changes in Lignin Synthesis Metabolites in Catalpa bungei ‘Jinsi’
Source: Metabolites. 2025 Jul 22;15(8):493. doi: 10.3390/metabo15080493 (PMC12388274; doi:10.3390/metabo15080493)

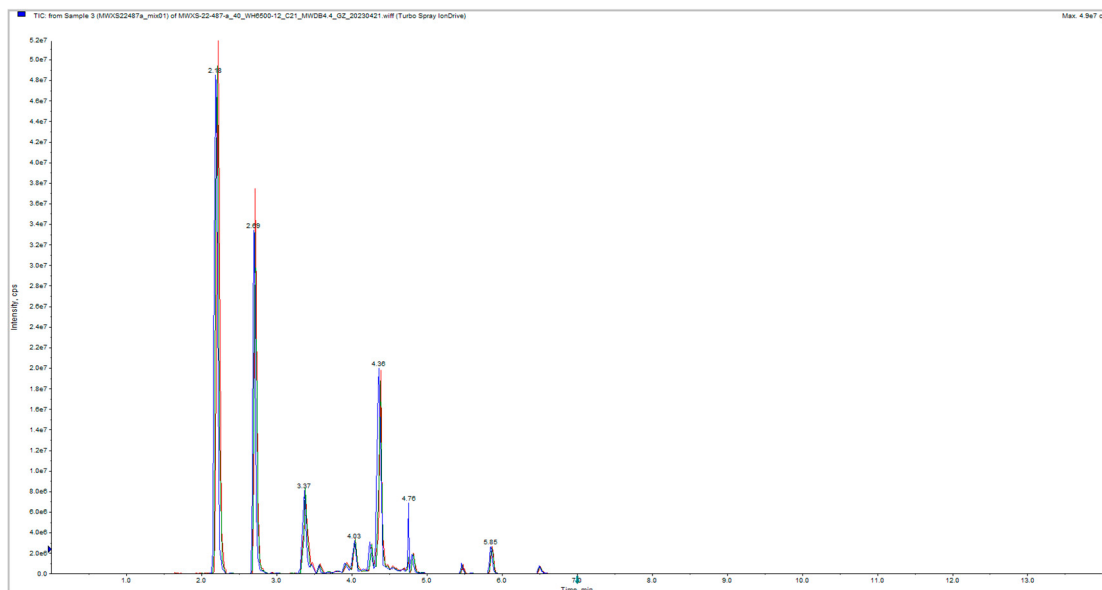

**Figure S1.** TIC overlap diagram of QC sample mass spectrometry detection.

Supplement: Supplementary file 1 [file metabolites-15-00493-s001.zip › metabolites-3677994-supplementary/Supplementary materials-Figure S1.pdf]
